# Resolution of ITD detection: stochastic vs. exquisite system

**DOI:** 10.1186/1471-2202-12-S1-P335

**Published:** 2011-07-18

**Authors:** Viacheslav A Vasilkov, Ruben A Tikidji-Hamburyan

**Affiliations:** 1 A.B.Kogan Research Institute for Neurocybernetics, Southern Federal University, Rostov-on-Don, 344090, Russia; 2 LSU Health Sciences Center, New Orleans, LA, 70112, USA

## 

Differences in arrival times of a sound at the two ears (Interaural Time Differences, ITDs) are utilized by mammals to determine the location of low-frequency sound source in the horizontal dimension [4]. Simple calculation shows that to achieve psychophysically revealed accuracy of sound localization, binaural auditory system have to detect ITDs in the order of just a few tens of microseconds. It has long been assumed that such remarkable temporal resolution is the result of ITD encoding by systematically organized arrays of fast neurons - “coincidence detectors” that receive inputs from the two ears via axons of variable length - “delay lines” and create a neural “map” of physiological ITDs. However, despite its wide acceptance, an increasing number of recent morphological and physiological studies in mammals report findings that can not be interpreted in the framework of this classical coincidence detector concept (reviewed in [2]). Therefore, neural mechanisms of the mammalian auditory system underlying high-resolution ITD detection are still not fully understood.

Here, we continue to consider an alternative ITD detection mechanism based on a population coding strategy [5], according to which ITDs are encoded by the relative “activity” levels within bilaterally located populations of binaural sluggish (slow) neurons. To implement studied ITD detection mechanism we employ biologically inspired computational model that simulates the behavior of two symmetric neural populations of the auditory system. Every single neuron model of each population has membrane time constant of >1 ms and receives the balanced excitatory-inhibitory (EI) synaptic inputs carrying ITD. The parameters of neuron [3] and synapse [1] models are turned to mimic pure trough-type activity of EI neurons, i.e., no (or minimal) responses occur when “ipsilateral” excitation and “contralateral” inhibition are near coincidence.

Our previous theoretical and modeling studies have shown that *precise* distribution (oppositely directed linear gradients) of the conductance value of excitatory and inhibitory synapses within each population allows the proposed model to detect ITD with submillisecond precision. It was also revealed that presence of jitter (related to the dynamic travel time deviation of ipsi- and contralateral signals) or neuronal noise from the physiological range does not have dramatic influence on ITD detection accuracy.

In this work, we focus on the further analysis of stochastic and noise processes in the auditory system and present a computational evidence indicating the necessity to reassess the role of stochasticity in ITD processing. We demonstrate that microsecond order resolution of ITD detection, based on the population coding mechanism, can be accomplished by the auditory system *without exquisite tunings* of synapses or/and axon lengths. It is also shown that biologically relevant levels of both intrinsic neuronal noise and random time jitter in the synaptic input underlie ITD detection acuity similar to that observed in the model with a precise as well as a random balance of excitatory and inhibitory synaptic inputs. Thus, the results suggest that the considered stochastic processes can play crucial role as complementary/replaceable sources of “perturbation” for the sluggish neuron based populations; that is needed for high-resolution ITD detection.
